# Treatment with organic manure inoculated with a biocontrol agent induces soil bacterial communities to inhibit tomato *Fusarium* wilt disease

**DOI:** 10.3389/fmicb.2022.1006878

**Published:** 2023-01-05

**Authors:** Tongtong Tang, Xing Sun, Qin Liu, Yuanhua Dong, Mingfang Zha

**Affiliations:** ^1^School of Biological Science and Food Engineering, Chuzhou University, Chuzhou, China; ^2^Institute of Soil Science Chinese Academy of Sciences, Nanjing, China; ^3^Institute of Soil Science Chinese Academy of Sciences, University of Chinese Academy of Sciences, Beijing, China; ^4^Chuzhou Agricultural and Rural Technology Extension Center, Chuzhou, China

**Keywords:** tomato *Fusarium* wilt, biocontrol agent, fertilizer treatment, rhizosphere bacterial communities, disease suppression

## Abstract

**Introduction:**

Organic manure, plant growth-promoting microorganisms, and biocontrol agents are widely used to sustainably control soil-borne diseases. However, how and whether organic manure inoculated with biocontrol agents alters soil microbiota and reduces disease severity is poorly understood.

**Methods:**

Here, we examined changes to the soil microbial community, soil properties, and incidence of *Fusarium* wilt disease in response to several fertilization regimes. Specifically, we studied the effects of inorganic chemical fertilization (*CF*), organic manure fertilization (OF), and *Erythrobacter* sp. YH-07-inoculated organic manure fertilization (BF) on the incidence of *Fusarium* wilt in tomato across three seasons.

**Results:**

BF-treated soils showed increased microbial abundance, richness, and diversity compared to other treatments, and this trend was stable across seasons. BF-treated soils also exhibited a significantly altered microbial community composition, including increased abundances of *Bacillus*, *Altererythrobacter*, *Cryptococcus*, and *Saprospiraceae*, and decreased abundances of *Chryseolinea* and *Fusarium*. Importantly, BF treatment significantly suppressed the incidence of Fusarium wilt in tomato, likely due to direct suppression by *Erythrobacter* sp. YH-07 and indirect suppression through changes to the microbial community composition and soil properties.

**Discussion:**

Taken together, these results suggest that *Erythrobacter* sp. YH-07-inoculated organic manure is a stable and sustainable soil amendment for the suppression of *Fusarium* wilt diseases.

## Introduction

High-intensity monoculture agriculture often leads to a high prevalence of soil-borne plant diseases, creating a serious threat to sustainable agricultural development. Of these soil-borne diseases, *Fusarium* wilt (*Fusarium oxysporum*) can be one of the most severe, and has the potential to infect a broad array of fruit and vegetable crops. In particular, tomato (*Solanum lycopersicum* L.), one of the most important and valuable vegetable crops worldwide, is susceptible to wilt caused by *F. oxysporum* f. sp. *lycopersici* (Walker, 1971; Armstrong and Armstrong, 1975).

Historically, control of *Fusarium* wilt relied primarily on chemical pesticides, such as methyl bromide and chloropicrin. However, although these chemicals can control pathogenic organisms, they can also destroy beneficial organisms. Additionally, many pesticide residues are harmful to animal and human health. Therefore, because chemical pesticide use is associated with safety and ecological risks (Gamliel et al., 2000; Fira et al., 2018; Syed Ab Rahman et al., 2018), it is of utmost importance to develop effective, safe, and sustainable control measures for soil-borne pathogens.

Various soil amendments have been found to alter rhizospheric microbial community composition, and through legacy effects can continue to alter these communities over time (Shen et al., 2013; Wang et al., 2015; Xiong et al., 2015; Liu H. et al., 2018). For example, the addition of organic manures has been found to control soil-borne pathogens *via* manipulation of the soil microbial community (Liu et al., 2022). The complex mechanism by which the addition of organic materials alters soil microbial communities has been extensively studied (Wei et al., 2011; Belén and Biosca, 2017; Busby et al., 2017; Neal and Glendining, 2019; Dai et al., 2022). One particularly important aspect of this complex interaction appears to involve the activity of plant growth-promoting rhizobacteria (PGPR).

To date, several PGPR have been successfully employed as both biopesticides and biostimulants across a wide variety of agricultural crops. PGPR promote plant growth through several mechanisms, including antibiotic, siderophore, and phytohormone production, nitrogen fixation, interspecific competition, and induced systemic resistance (ISR; Lutz et al., 2004; Pliego et al., 2008; Sarma et al., 2015; Backer et al., 2018; Thomloudi et al., 2019). Because of the diversity of plant growth-promoting effects exhibited by PGPR, research is still underway to fully characterize and realize the potential of PGPR as agricultural agents.

In our previous study, we reported that the application of *Erythrobacter* sp. YH-07, a PGPR isolated from Yellow Sea mud, inhibited tomato *Fusarium* wilt and altered the structure of the soil microbial community (Tang et al., 2020). However, we also found that the application of *Erythrobacter* sp. YH-07 reduced the overall diversity of soil microbes. In order to combat this loss of diversity, here we conducted a three-season cultivation experiment to test the ability of three fertilization regimes to control *Fusarium* wilt of tomato: inorganic chemical fertilization (*CF*), organic manure fertilization (OF), and *Erythrobacter* sp. YH-07-inoculated organic manure fertilization (BF). We examined the resultant changes to the soil microbiota using high-throughput sequencing. This experiment was undertaken to evaluate the relationship between fertilizer management, soil microbial population dynamics, and disease suppression.

## Materials and methods

### Experimental design

The soil used for these experiments was collected from vegetable production greenhouses known to harbor tomato wilt pathogens, located at the Vegetable Research Institute of Jiangsu Academy of Agricultural Sciences, Nanjing, China. The experimental soil contained 15.02 g kg^−1^ SOC, 1.14 g kg^−1^ total N, 82.78 mg kg^−1^ Alkaline-N, 1.01 g kg^−1^ total P, 18.74 mg kg^−1^ available P, 8.79 g kg^−1^ total K, and 135.63 mg kg^−1^ available K. The three-season cultivation experiment was performed from March 14th to August 30th in 2019, 2020, and 2021. The three fertilization treatments were as follows: (1) soil amended with chemical fertilizer (*CF*), (2) soil amended with organic manure (OF), and (3) soil amended with organic manure inoculated with *Erythrobacter* sp. YH-07 (BF). The organic manure consisted of cow dung and straw stalk composted at 30°C–70°C for at least 30 days. The inoculated manure was produced by secondary fermentation according to the modified method of Liu Y. et al. (2018). Briefly, to obtain the liquid fermentation inoculant, the second-generation pathogen-antagonistic PGPR *Erythrobacter* sp. YH-07 was inoculated onto nutrient agar (NA) culture medium and incubated in a temperature-controlled incubator at 30°C and 170 rpm for 48 h until a final concentration of >5 × 10^9^ CFU g^−1^ was reached. Subsequently, 1,000 ml of the liquid fermentation inoculant was added to 50 kg of cow manure and subjected to solid fermentation for 72 h, with the entire pile manually turned over with a spade once per day. After secondary fermentation, the YH-07 cell concentration in the cow manure compost was found to be >1 × 10^8^ CFU g^−1^ dry weight. The plate counting method was used to assess the concentration.

For each treatment, either the designated fertilizer or organic manure was homogenously mixed with soil in a 1:100 ratio and added to sterilized pots (25 cm bottom diameter, 37 cm depth, ultraviolet light was used to sterilize the pots for 48 h). Each treatment consisted of three replicates, and each replicate contained five pots. One tomato (‘Hezuo 908’, *Fusarium* wilt susceptible) seedling, at the four true leaves stage, was planted in each pot. Tomato seedlings were grown in a greenhouse (located at the Institute of Soil Science, Chinese Academy of Sciences, Nanjing, China) under controlled conditions, with the temperature and relatively humidity maintained at 28°C–32°C and 58%–70%, respectively. Tomato seedlings were monitored daily for the appearance and severity of *Fusarium* wilt disease. Disease symptoms typically manifested themselves ~3 weeks after planting (April 14th 2019, April 18th 2020, April 17th 2021).

### Soil sampling and tomato disease assay

Soil sampling was performed in July of each cropping season, according to the modified method of Liu H. et al. (2018). Briefly, we collected three haphazard soil cores (0–10 cm in depth) from each pot using a 1.5-cm diameter sore corer. The three samples from each replicate were mixed to form a composite, resulting in three samples per treatment. Each soil sample was sieved through a 2-mm sieve and thoroughly homogenized. One portion of each sample was air-dried for 4 days for soil physicochemical analysis, and the rest was stored at −80°C for subsequent DNA extraction. All soil chemical properties were determined according to the method of Bao (2010).

Disease incidence (DI) was calculated as the percentage of infected plants within the total population of plants.


$$\text{DI }=\sum \left(n_{i}\times i\right)/\left(N\times i_{\max}\right)\times 100\%$$

**n**_**i**_ is the number of plants with disease grade i, **i** is the plant disease grade, **i**_**max**_ is the highest disease grade, **N** is the total number of plants per treatment.

The severity of the tomato wilt disease was ranked according to five different grades: 0 indicated non-symptomatic; 1 indicated <25% incidence of wilting leaves; 2 indicated between ≥25 and < 50% incidence of wilting leaves; 3 indicated between ≥50 and < 75% wilting leaves; and 4 indicated between ≥75 and 100% wilted leaves (or death).

Logit model [log DI/(1-DI)] was used to transform the incidence data before one-way analysis of variance (ANOVA) was performed.

### Soil DNA extraction, PCR amplification, and Illumina sequencing

In order to determine whether disease suppression could be attributed to the biotic rather than abiotic properties of the soil, we performed a soil suppressiveness assay based on the method of Mendes et al. (2011), with some modifications. Genomic DNA was extracted from a 0.5-g subsample of each composite soil mixture using the MoBioPowerSoil DNA Isolation Kit (Mo Bio Laboratories Inc., Carlsbad, CA, United States), according to the manufacturer’s instructions. Genomic DNA concentration and purity were measured using a NanoDrop 2000 spectrophotometer (NanoDrop Technologies, Wilmington, DE, United States). The V4 hypervariable regions of the bacterial 16S rRNA gene was amplified using the following primers: 520F (5′-AYTGGGYDTAAAGNG-3′) and 802R (5′-TACNVGGGTATCTAATCC-3′). Primer pairs were modified for sequencing by adding the forward and reverse Illumina Nextera adapters, a 2-bp “linker” sequence, a unique 7-bp barcode sequence at the 5′ end of the forward primer, and a linker sequence at the 5′ end of the reverse primer. PCR was performed following previously published amplification specifications (Liu et al., 2016). Briefly, 25 cycles were performed to amplify the bacterial templates. Subsequently, the PCR products were purified using a PCR Purification Kit (Axygen Bio, Union City, CA, United States) and pooled in equimolar concentrations of 10 ng ml^−1^ prior to sequencing. Both amplification and sequencing of the 16S genes were performed at Personal Biotechnology Co., Ltd. (Shanghai, China) on an Illumina MiSeq instrument (Illumina Inc., San Diego, CA, United States). All sequences were deposited in the NCBI Sequence Read Archive database^1^ under the accession number RPJNA859027.

### Bioinformatics analyses

Quality control and annotation of the raw sequences were performed according to Liu et al. (2016). A total of 36,473 16S rRNA gene sequences from each sample were randomly selected for further analysis of the microbial community structure. The Sobs, Shannon, Chao1, abundance-based coverage estimator (ACE), and the Coverage index were calculated using the operational taxonomic unit (OTU) results (Zhuykova et al., 2022). Sobs represents the observed richness of the community, the Shannon index describes the disorder and uncertainty associated with individual species within a community (the higher the uncertainty, the higher the diversity), the Chao1 richness index estimates the number of OTUs within a sample (the larger the Chao1 value, the higher the diversity), the ACE index represents the richness and evenness of the community (larger ACE values represent higher richness and evenness), and the Coverage index is indicative of the Good’s coverage.

A non-metric multidimensional scaling (NMDS) analysis, based on the Bray-Curtis distance, was used to compare microbial community composition between soil samples. Specifically, NMDS is a data analysis method that simplifies the research objects (samples or variables) in multi-dimensional space to low-dimensional space for positioning, analysis, and classification, while retaining the original relationship between objects. The correlation between microbial community composition and soil characteristics was estimated using a Mantel test with the R (Version 3.2.2) software package “vegan.” A Mantel test is a nonparametric statistical method to test the correlation (Spearman rank correlation coefficient, etc.) between a community distance matrix (such as a UniFrac distance matrix) and an environmental variable distance matrix (such as a difference matrix of pH, temperature, or geographical location). The correlation between selected genera of rhizospheric microbes (relative abundance >0.1%) and *Fusarium* wilt disease incidence was estimated using Pearson’s correlation coefficient.

In order to characterize the relationships among soil microbial genera, soil parameters, and environmental variables, redundancy analysis (RDA) was carried out using Canoco 5.0 software (Microcomputer Power Inc., United States). RDA is a principal component analysis (PCA) constrained by environmental factors, which can be used to create an intuitive diagram reflecting the relationship between sample distribution and environmental factors. Prior to correlational analysis, the environmental factors can be screened and those with minimal multicollinearity can be retained for subsequent research. Variance inflation factor (VIF) analysis was used for environmental factor screening, using a subset of environmental variables. Soil properties were selected using the envfit and VIF functions, and the factors with *p* > 0.05 or VIF > 20 were removed.

### Statistical analyses

To determine statistically significant differences (*p* < 0.05) between treatments, one-way ANOVA and Duncan’s multiple range test was performed at the conclusion of each bioassay. Statistical analyses were performed using the IBM SPSS 20.0 software program (IBM Corporation, New York City, NY, United States).

## Results

### Effect of fertilization treatments on disease incidence

**Figure 1:**
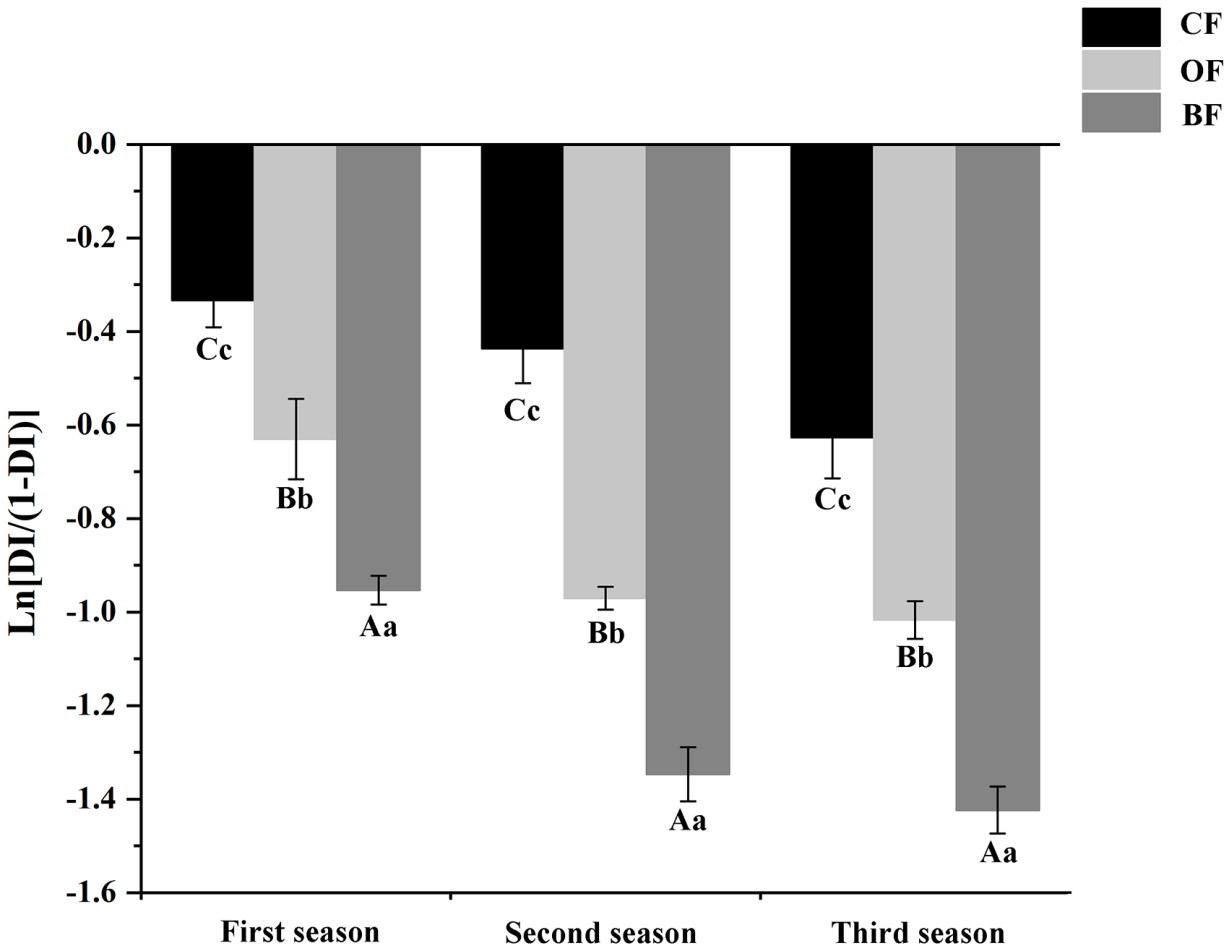
Effects of different fertilization treatments on disease incidence of *Fusarium* wilt disease of tomato. *CF*: inorganic chemical fertilizer; OF: organic manure; BF: organic manure inoculated with a biocontrol agent. Different letters mean differ significantly (capital letters and small letters represent differences of within-group and between-group respectively; Duncan’s test, *p* < 0.05).

The incidence trend of *Fusarium* wilt was similar across all three seasons, but the disease incidence was significantly lower (*p* < 0.05) in the third season than the first season (Figure 1). In different seasons, the application of OF and BF significantly (*p* < 0.05) reduced disease incidence compared to the *CF* treatment. These results indicate that both the application of organic manure and the addition of the microbial biocontrol agent were able to effectively control *Fusarium* wilt in tomato.

### Effect of fertilization treatments on soil characteristics

The soil physicochemical characteristics were similar across all three seasons, although the different fertilization treatments altered these characteristics (Table 1). Both the application of OF and BF significantly increased the content of several chemical constituents compared to the *CF* treatment. In particular, the BF-treated soil had significantly higher soil organic carbon (SOC), alkaline N, and available potassium (AK) contents compared to the other treatments.

**Table 1:** physicochemical properties of soil samples under the different fertilization treatments.

	First season			Second season			Third season		
	*CF*	OF	BF	*CF*	OF	BF	*CF*	OF	BF
SOC (g kg^−1^)	15.16 ± 0.05 c	18.36 ± 1.35 b	21.76 ± 0.28 a	15.31 ± 0.24 c	17.06 ± 0.81 b	22.09 ± 0.57 a	15.58 ± 0.30 c	19.75 ± 0.39 b	21.54 ± 0.54 a
TN (g kg^−1^)	1.29 ± 0.08 b	1.62 ± 0.09 a	1.41 ± 0.04 b	1.39 ± 0.11 a	1.52 ± 0.07 a	1.44 ± 0.04 a	1.36 ± 0.05 a	1.49 ± 0.14 a	1.43 ± 0.01 a
Alkeline-N (mg kg^−1^)	93.74 ± 4.14 c	109.83 ± 1.35 b	125.48 ± 3.25 a	99.12 ± 5.15 c	111.32 ± 2.96 b	128.96 ± 1.76 a	105.21 ± 8.59 b	109.76 ± 1.60 b	130.49 ± 4.50 a
TP (g kg^−1^)	1.13 ± 0.11 c	1.67 ± 0.04 b	1.88 ± 0.04 a	1.21 ± 0.06 b	1.72 ± 0.08 a	1.83 ± 0.10 a	1.43 ± 0.08 c	1.64 ± 0.13 b	1.90 ± 0.05 a
Available P (mg kg^−1^)	19.69 ± 2.31 c	31.14 ± 1.59 b	41.89 ± 0.94 a	22.77 ± 4.68 b	20.29 ± 0.96 b	37.90 ± 1.51 a	28.06 ± 1.65 c	32.93 ± 0.60 b	37.68 ± 0.47 a
TK (g kg^−1^)	9.12 ± 0.11 b	9.77 ± 0.17 a	9.67 ± 0.24 a	9.24 ± 0.10 b	9.84 ± 0.07 a	9.73 ± 0.13 a	9.28 ± 0.08 b	9.90 ± 0.18 a	9.88 ± 0.32 a
Available K (mg kg^−1^)	147.16 ± 6.56 c	197.87 ± 8.83 b	232.54 ± 9.36 a	153.22 ± 19.75 b	222.43 ± 6.39 a	241.62 ± 10.75 a	187.16 ± 6.29 c	219.67 ± 10.57 b	243.37 ± 4.11 a

CF, inorganic chemical fertilizer; OF, organic manure; BF, organic manure inoculated with a biocontrol agent. The values are the means of five replicates (means ± SD), Values followed by different letters differ significantly (Duncan’s test, *p* < 0.05).

### Microbial community composition

**Figure 2:**
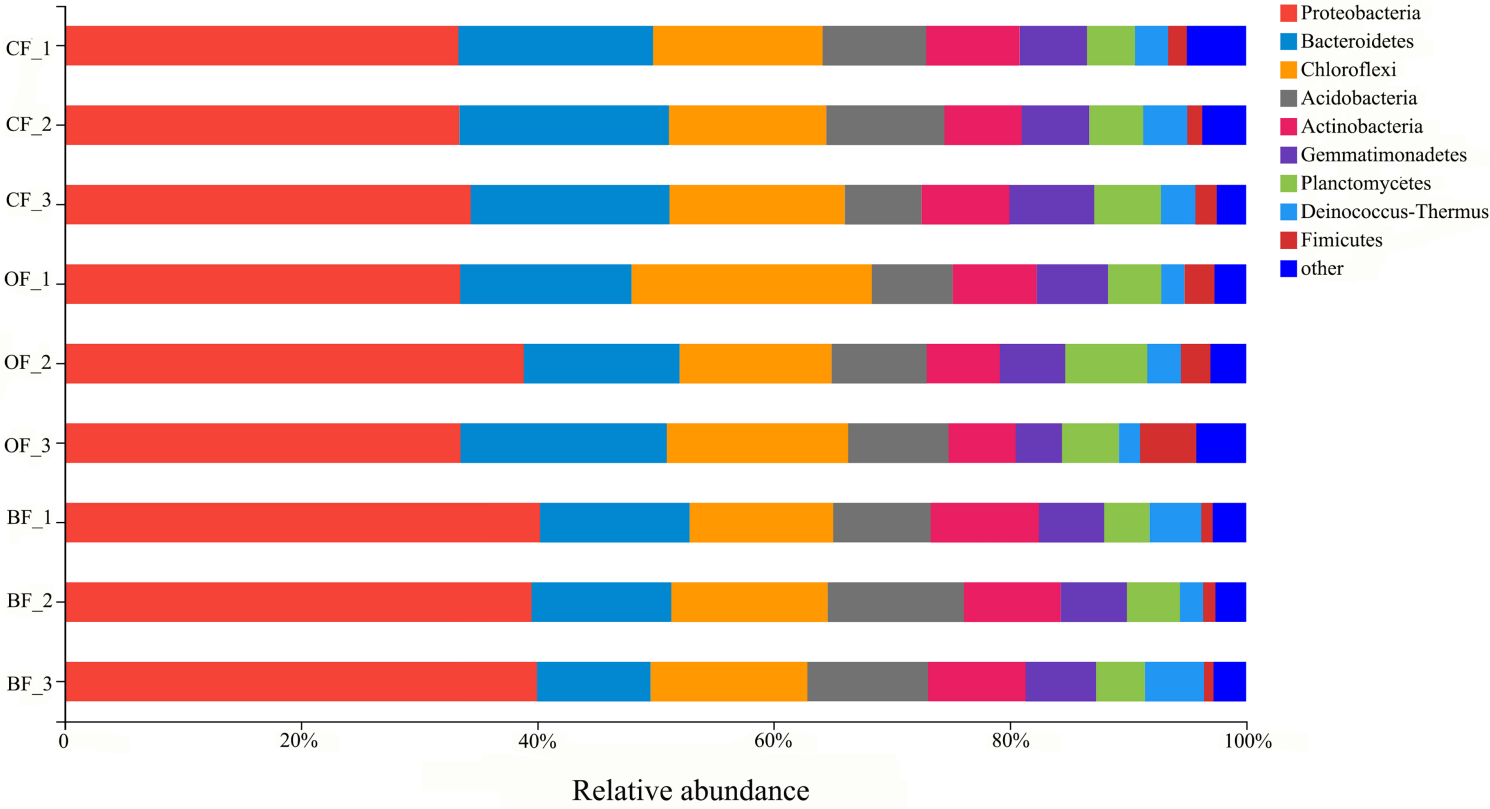
The relative abundance of bacterial phyla from different fertilizer treatments. *CF*: inorganic chemical fertilizer; OF: organic manure; BF: organic manure inoculated with a biocontrol agent. Number 1, 2, and 3 mean three different seasons.

A total of 287,955 16S rRNA sequences were grouped into 5,086 bacterial OTUs at the 97% similarity cut-off level. Additionally, bacterial sequences were classified into a total of 43 phyla. The 10 most dominant phyla were Proteobacteria, Bacteroidetes, Chloroflexi, Acidobacteria, Actinobacteria, Gemmatimonadetes, Planctomycetes, Deinococcus-Thermus, and Firmicutes (Figure 2). In particular, Proteobacteria displayed a higher relative abundance in the BF and OF treatments than in the *CF* treatment, but Firmicutes displayed a higher relative abundance in the *CF* treatment than in the BF treatment.

**Figure 3:**
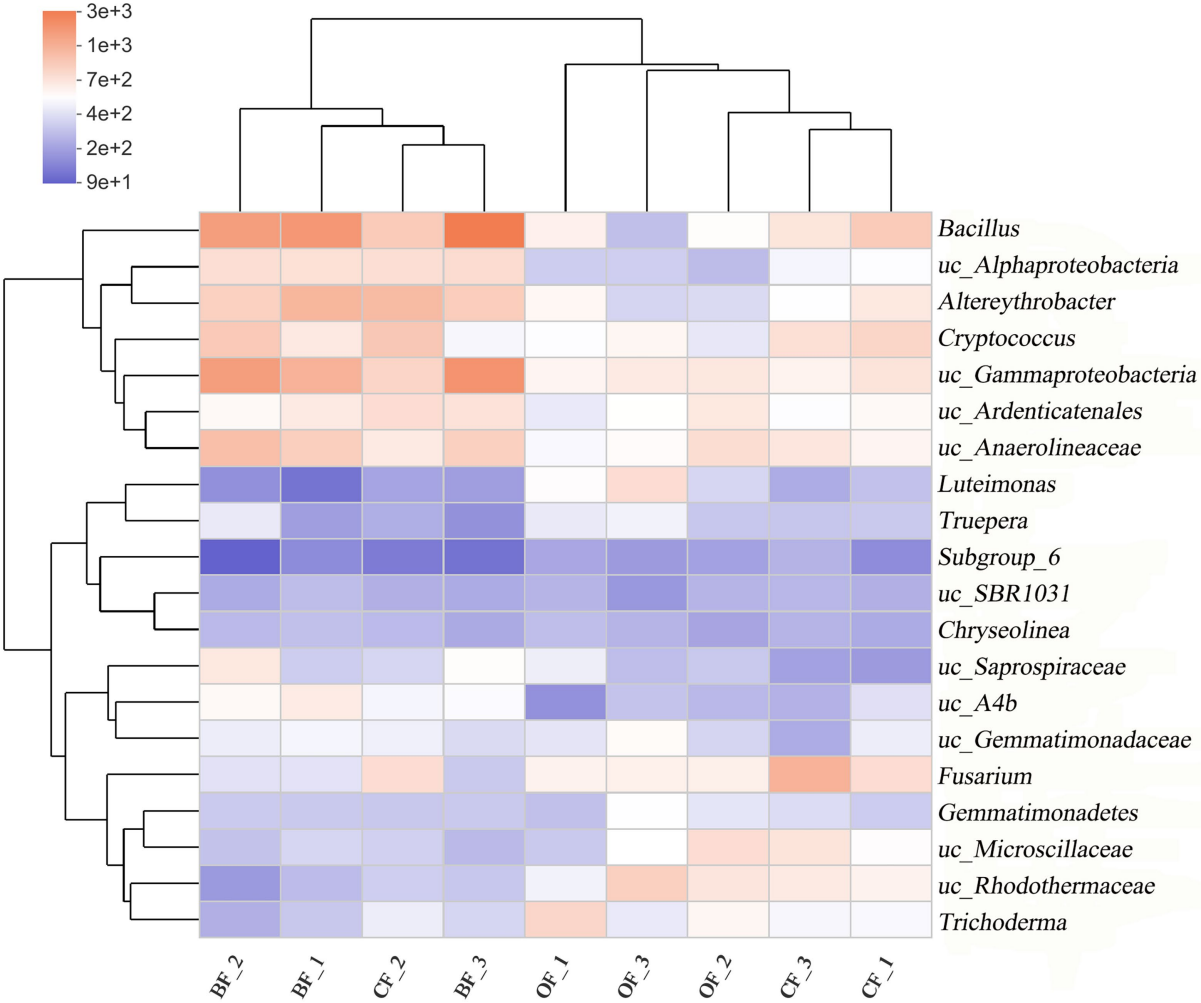
Microbial community composition at the genus level of different fertilization management programs. Number 1, 2, and 3 mean three different seasons. uc: unclassified.

A heatmap and phylogenetic clustering tree were created to visualize the genus-level microbial community composition of the different fertilization treatments across seasons (Figure 3). Overall, the microbial community composition of the BF-treated soil was markedly different from that of either the OF- or *CF*-treated soil. Additionally, the abundance of soil microbes was different across the three planting seasons, even under the same treatment.

### Microbial community structure, abundance, and diversity

Both OF and BF treatment significantly altered species richness and diversity (Table 2). BF-treated soil displayed a significantly higher (*p* = 0.031) Chao1 index compared to *CF*-treated soil. The ACE index showed the same trend (*p* = 0.025), although the difference between BF and OF was not significant (*p* = 0.755). In contrast, the OF treatment displayed a significant decrease in Shannon index (*p* = 0.033). The Sobs index showed no significant difference among the three treatments and all treatments had the same Good’s coverage.

**Table 2:** Soil microbial alpha-diversity indices across fertilization treatments.

	Chao1	Ace	Shannon	Sobs	Coverage
*CF*	3038.84 ± 71.48 b	3077.74 ± 50.32 b	6.31 ± 0.03 a	2318.33 ± 61.82 a	0.98 ± 0.00 a
OF	3350.53 ± 33.44 a	3347.86 ± 62.53 a	6.15 ± 0.01 b	2407.33 ± 45.17 a	0.98 ± 0.00 a
BF	3685.67 ± 512.25 a	3689.88 ± 517.46 a	6.39 ± 0.17 a	2641.00 ± 350.35 a	0.98 ± 0.00 a

CF, inorganic chemical fertilizer; OF, organic manure; BF, organic manure inoculated with a biocontrol agent. The values are the means of three replicates (means ± SD), Values followed by different letters differ significantly (Dunnett’s test, *p* < 0.05).

**Figure 4:**
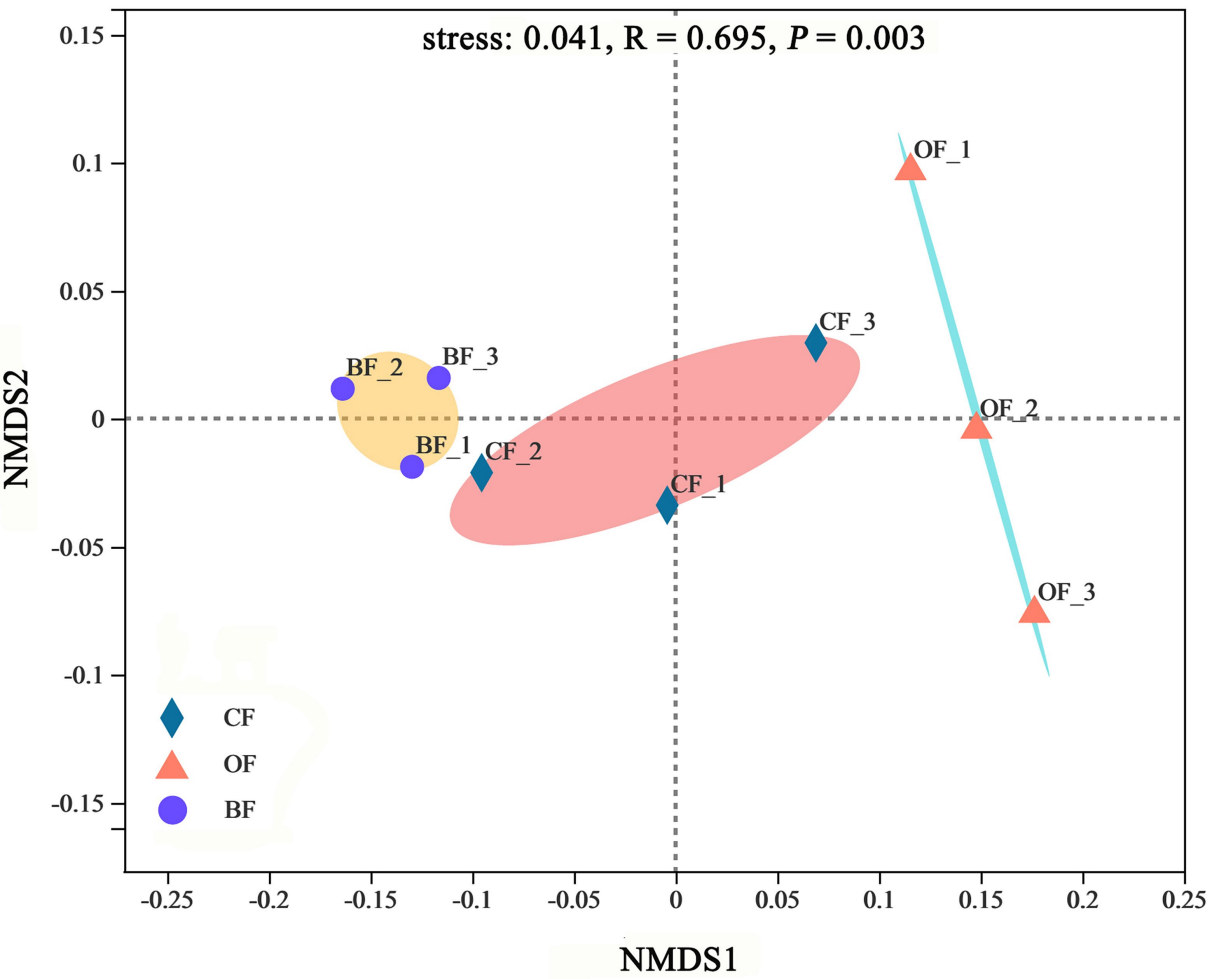
Non-metric multidimensional scaling (NMDS) of microbial community based on Bray-Curtis distance metric among different fertilization management programs. Number 1, 2, and 3 mean three different seasons.

According to the NMDS analysis, the microbial communities were significantly different between treatments (stress = 0.04, *p* < 0.01), producing 3 highly-supported clusters (Figure 4). Additionally, microbial communities within treatments were more similar to each other than those from different treatments.

### Effect of soil characteristics on microbial communities

**Figure 5:**
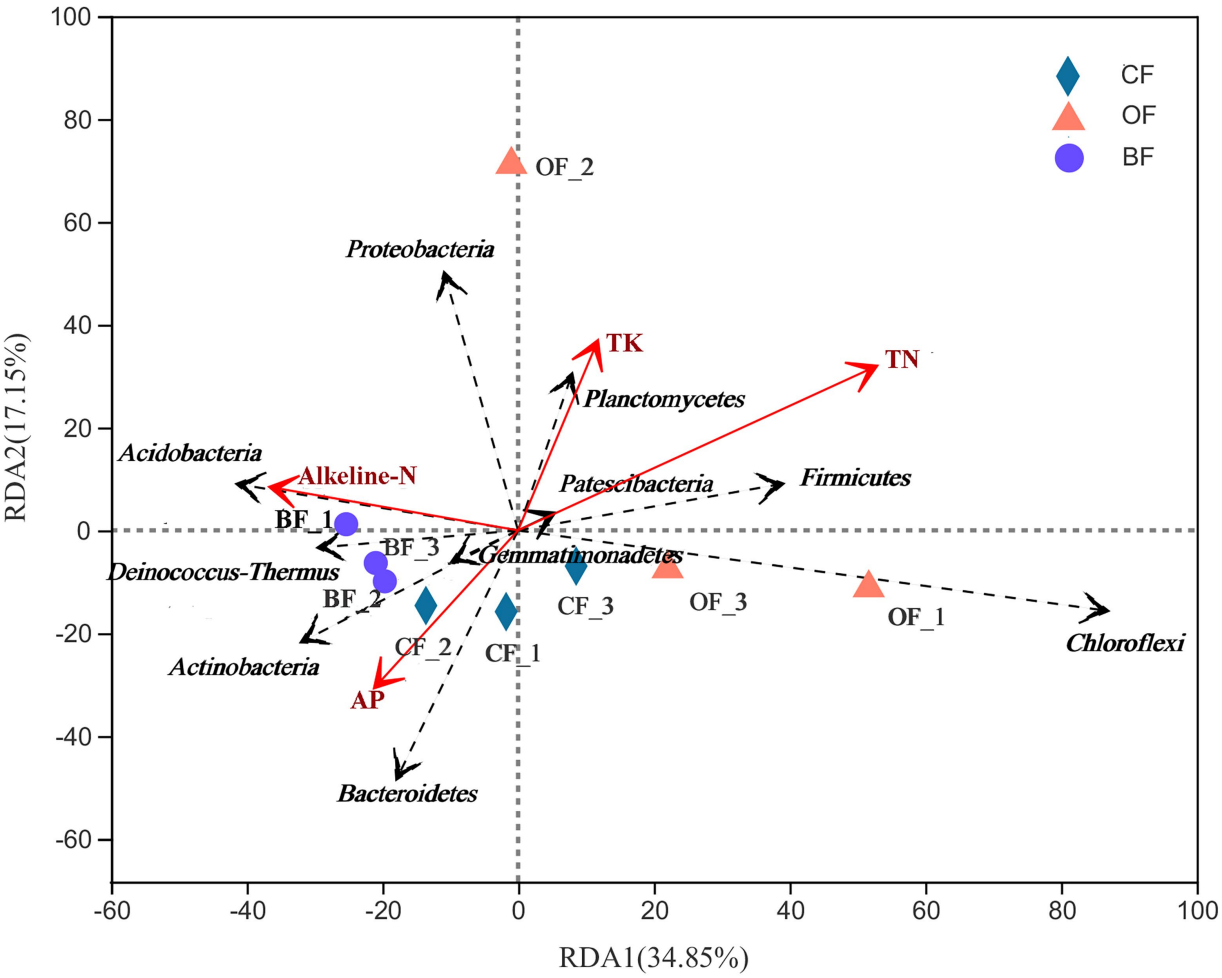
Redundancy analysis (RDA) of the relationship between the analyzed soil samples and environmental variables. Number 1, 2, and 3 mean three different seasons.

Overall, differences in soil characteristics were found to be related to differences in microbial community structure. Creation of a UniFrac distance matrix revealed that selected soil chemical properties (VIF values of SOC, TP, and AK were higher than 20 and removed) were significantly correlated with microbial genera (*r* = 0.48, *p* = 0.02). Additionally, the RDA indicated that the two components explained 51.0% of the total variation (Figure 5). The first component (RDA1) explained 34.9% of the variation, effectively separating the BF treatment from the others. The microbial community of BF-treated soils was dominated by Gemmatimonadetes, Deinococcus-Thermus, Actinobacteria, and Acidobacteria, which were related to the content of alkaline-N. The microbial community of *CF*-treated soils was dominated by Bacteroidetes, which was related to the content of available phosphorus (AP). The microbial community of OF-treated soils was dominated by Proteobacteria, Planctomycetes, and Chloroflexi.

## Discussion

*Fusarium* wilt disease is an economically important fungal disease of tomato. Applications of organic fertilizer and bio-organic fertilizer significantly suppressed tomato disease and improved fruit yield compared to the chemical fertilizer. In our previous study, we reported that the application of the biocontrol PGPR *Erythrobacter* sp. YH-07 to newly-established tomato plantations effectively suppressed *Fusarium* wilt by stabilizing the culturable bacterial community composition (Tang et al., 2020). In this study, we sought to expand on these findings by examining whether organic manure inoculated with *Erythrobacter* sp. YH-07 (BF) can effectively suppress *Fusarium* wilt. We also examined changes in the microbial community and soil properties in order to ascertain the mechanism of disease suppression.

Overall, the application of BF significantly suppressed *Fusarium* wilt in tomato, compared to *CF*. These results are in agreement with previous studies suggesting that organic treatments can be used to effectively control disease caused by soil-borne pathogens (Cheng et al., 2021). Moreover, the BF treatment was more efficacious than OF alone, which is in line with previous results indicating that organic matter inoculated with biocontrol microbes can control *Fusarium* wilt more effectively than application of either component alone (Liu H. et al., 2018). We hypothesize that the microbial communities determined the significant difference in disease incidence.

The application of organic manure (OF and BF) tended to increase microbial abundances compared to the inorganic fertilizer treatment, in agreement with a previous report (Li et al., 2022), who showed more diverse microbial communities were found in rhizosphere soil. Highly diverse microbial communities tend to create an environment which is hostile to pathogens, often due to resource competition (van Elsas et al., 2012). We suggest that the expected increase in soil microbial richness and diversity resulting from continuous BF application may contribute to effective control of *Fusarium* wilt in long-term field situations. Specifically, we observed higher microbial species richness (Chao1) in BF-treated soils, consistent with reports in banana (Shen et al., 2013), watermelon (Ling et al., 2014), and cucumber (Qiu et al., 2012). Research by Dey et al. (2012) indicated a negative correlation between microbial diversity and soil-borne disease incidence, but we found no correlation between microbial diversity and incidence of *Fusarium* wilt. Together, these findings indicated that not only the higher microbial diversity, but also the microbial communities, determined the difference in disease incidence. Future studies using a time series in long-term experiments would be helpful for disentangling the relationships between microbiota and *Fusarium* wilt disease.

Soil-borne disease suppression can often be attributed to complex changes in microbial community composition, particularly to changes in the abundance of specific microbes, such as Proteobacteria, Firmicutes, and Actinobacteria (Raaijmakers et al., 2009; Mendes et al., 2011). Across all soils in this study, Proteobacteria, Acidobacteria, Actinobacteria, Bacteroidetes, and Firmicutes were the predominant bacterial phyla (Figure 2), consistent with previous studies on soil microbial communities (Bonilla et al., 2012). In addition, microbial community composition significant differences between BF- and *CF*-treated soils, indicated that amendment type was the most influential factor on microbial community composition. Many studies have confirmed organic manure soil amendment to change the microbial community composition (Uroz et al., 2013; Hartmann et al., 2015; Shen et al., 2015; Sun et al., 2015) Overall, treatment with BF was the greatest driver of microbial community composition, leading to pathogen inhibition. In accordance with the results of organic manure inoculated with biocontrol microbes act almost universally to inhibit invasion by a variety of pathogens (Bonilla et al., 2012; Shen et al., 2013; Almario et al., 2014).

According to our Spearman correlation analysis (Table 3), the incidence of *Fusarium* wilt was correlated with changes in microbial community composition, with specific taxa displaying particularly strong correlations. Among these highly correlated taxa, the abundance of *Fusarium oxysporum*, the pathogen responsible for *Fusarium* wilt, was significantly reduced in BF-treated soils, compared to *CF*-treated soils (Table 3). Although pathogenic and non-pathogenic *Fusarium* species were not individually quantified in this study, we found a significant positive correlation between the abundance of *Fusarium* and disease incidence, suggesting that the abundance of *Fusarium* could be used as an indicator of the pathogenicity of *Fusarium* species generally. Previous work has found that bioorganic fertilization effectively reduced the abundance of *Fusarium* in cucumber and banana (Qiu et al., 2012; Shen et al., 2015), supporting the notion that organic manure application controls plant diseases through reducing the abundance of pathogenic organisms (Bonanomi et al., 2010).

**Table 3:** Spearman’s rank correlation coefficient between rhizosphere abundant genus and disease incidence.

	Disease incidence
*Bacillus*	−0.63**
*uc__Alphaproteobacteria*	−0.78**
*Altererythrobacter*	−0.56*
*Cryptococcus*	−0.47*
*uc__Gammaproteobacteria*	−0.43*
*uc__Saprospiraceae*	−0.47*
*Fusarium*	0.53*
*Chryseolinea*	0.41*
*Trichoderma*	0.56*

Statistical significance at *p < 0.05 and **p < 0.0.

We also found that the taxa *Chryseolinea* and *Trichoderma* were significantly positively correlated with *Fusarium* wilt incidence. *Chryseolinea*, belonging to Bacteroidetes, was discovered by German scientists (Kim et al., 2013), and Chinese scientists have isolated similar strains from soil samples (Wang et al., 2018). However, the ecological and pathological significance of *Chryseolinea* is poorly understood. Perhaps these bacteria support pathogen infestation and disease progression through secretion or degradation of soil organic matter. Studies have confirmed that members of the genus *Trichoderma* act as biocontrol agents and provide protection against soil-borne pathogens (Ent et al., 2009). Previous studies suggest that this may be related to the citric acid and fumaric acid secreted into the soil by plants infected with pathogens, which stimulates the chemotaxis response of biocontrol agents (Liu H. et al., 2018). However, in this study, the relative abundance of *Trichoderma* was positively correlated with tomato wilt disease incidence.

In contrast, several taxa were significantly negatively correlated with *Fusarium* wilt incidence, including *Bacillus*, *Altererythrobacter*, *Cryptococcus*, and *Saprospiraceae*. Some species of *Bacillus* are already used as biocontrol agents against a wide range of fungal pathogens, including *Rhizoctonia cerealis*, *Botrytis cinerea*, *Penicillium digitatum*, and *P. italicum* (Chen et al., 2020; Samaras et al., 2021; Yi et al., 2022). BF-treated soil also displayed a high abundance of *Altererythrobacter*, which is consistent with previous studies showing stable proliferation of the *Erythrobacter* YH-07 strain (which belongs to Altererythrobacter) after soil inoculation (Tang et al., 2020). *Cryptococcus* are antagonistic toward pathogenic microbes through several mechanisms, including competition for space and nutrients, production of hydrolytic enzymes, induction of disease resistance in plants, and biofilm formation (Bautista-Rosales et al., 2014; Schisler et al., 2014). To our knowledge, there are no reports of *Saprospiraceae* having specific biocontrol activity. However, because the species is negatively correlated with *Fusarium* wilt disease incidence, we suggest that these are potentially novel biocontrol agents.

Soil properties can also be important for the suppression or proliferation of soil-borne diseases. We found that BF-treated soils had significantly higher SOC, alkaline-N, AK, and AP contents, as well as lower disease incidence, compared to *CF*-treated soils. Previous studies have reported that high soil organic matter (OM) content was negatively correlated with *Fusarium* abundance in a potato monoculture system (Liu et al., 2014) and that high soil AP was negatively correlated with Fusarium wilt disease incidence in banana (Shen et al., 2015). It may be that improved soil properties promote plant health, thereby enhancing plant resistance to disease. However, we found that these soil characteristics by themselves were not sufficient to reduce disease incidence in this study. We suggest that the inhibition of disease caused by application of organic manure inoculated with biocontrol microbes may be related to the feedback loop between soil properties and microbial communities. The altered soil environment may alter the soil microbiome, and vice versa, to contribute to disease suppression and plant health. We suspect that the main reason is that biocontrol microorganisms in the organic manure are able to colonize the rhizosphere soil of plants and secrete signaling substances to achieve biocontrol. However, the presence and activity of signaling substances requires further validation.

## Conclusion

The enhancement of disease suppression through application of a variety of organic and biological soil amendments has been widely studied (Mazzola, 2004; Bonilla et al., 2012). We found that organic cow manure-based fertilizer inoculated with a biocontrol microbe (BF) was able to effectively suppression *Fusarium* wilt in tomato. BF treatment also resulted in changes to the soil microbial community composition and soil chemical properties. In fact, alterations in the microbial community and soil chemical properties worked synergistically with the biocontrol agent to further suppress *Fusarium* wilt disease incidence. Overall, BF treatment acted as the primary driver of soil microbial community composition by increasing bacterial abundance, richness, and diversity, suggesting that this treatment may contribute to the induction of a broad-spectrum disease resistance. Specifically, BF treatment increased the abundances of *Bacillus*, *Altererythrobacter*, *Cryptococcus*, and *Saprospiraceae*, and decreased the abundances of *Fusarium* and *Chryseolinea*. While some of the microbes which were found to increase in abundance in response to BF treatment are known biocontrol agents, others, namely *Saprospiraceae*, are potentially novel and may provide a new biocontrol resource. This study provides insights into the role of organic manures and biocontrol microbes in inhibiting soil-borne diseases and creating healthy soil microbial communities.

## Data availability statement

The data presented in the study are deposited in the NCBI repository (https://www.ncbi.nlm.nih.gov/genbank/), accession numbers SRR20219027, SRR20219028, SRR20219029, SRR20219030, SRR20219031, SRR20219032, SRR20219033, SRR20219034 and SRR20219035.

## Author contributions

TT and XS contributed to conception and design of the study and performed the statistical analysis. TT organized the database. QL wrote the first draft of the manuscript. YD and MZ wrote sections of the manuscript. All authors contributed to the manuscript revision, read, and approved the submitted version.

## Funding

This research was supported by the National Key Research and Development Program of China (no. 2017YFD0200604), National Natural Science Foundation of China (no. 31400464), Anhui Natural Science Foundation (no. 2108085MC87) and Research Startup Foundation of Chuzhou University (no. 2020qd35).

## Conflict of interest

The authors declare that the research was conducted in the absence of any commercial or financial relationships that could be construed as a potential conflict of interest.

## Publisher’s note

All claims expressed in this article are solely those of the authors and do not necessarily represent those of their affiliated organizations, or those of the publisher, the editors and the reviewers. Any product that may be evaluated in this article, or claim that may be made by its manufacturer, is not guaranteed or endorsed by the publisher.

## Footnotes

^1^ http://www.ncbi.nlm.nih.gov/Traces/sra
